# Spastic and Syphilitic Spinal Paralysis

**Published:** 1902-10-18

**Authors:** 


### SPASTIC AND SYPHILITIC SPINAL PARALYSIS.

The managers of the Post-graduate College connected with the West London Hospital were fortunate in being able to obtain an opening lecture from so celebrated an authority on nerve diseases as Dr. Erb, Professor of Clinical Medicine at the University of Heidelberg. Professor Erb devoted the occasion to a discussion of two forms of spinal paralysis which he has been able to associate with certain definite changes in the spinal cord, namely, spastic and syphilitic spinal disease. In spastic spinal paralysis there is present, as the essential change, a sclerosis of the posterior segments of the lateral spinal tracts, and particularly the pyramidal tracts, more or less uncomplicated and exclusive. The complaint is on the whole a rare one, much rarer than tabes. It usually begins slowly and insidiously, rarely in a more acute fashion, with some sense of weight, dragging, and slight feebleness in one or the other leg, without pain, or at most only pain or fatigue after prolonged exertion, without, or with only slight, transient paresthesia of the legs, and without any other symptom except, perhaps, some slight backache. The condition progresses slowly, the legs become stiffer and heavier, the gait more laboured, dragging, and distinctly spastic. There may be occasionally muscular cramps and contractions of the legs but nothing more, and even after years the characteristic symptom triad may remain uncomplicated. This symptom-triad may be thus described :—First there is a certain weakness and awkwardness of movement of the legs, very slight and without any ataxy. Second, and much more prominent, are the spasm and rigidity of the muscles, ranging from slight elastic resistance during passive movement to marked, stiff contracture, temporarily increased by energetic muscular action, necessitating forced extension of the legs; and, thirdly, well marked exaggerations of the tendon reflexes, with involuntary clonus of the foot while the patient is sitting. A fourth sign has been added to this triad by Babinski, namely the plantar reflex, consisting of a slow dorsiflexion of the great toe when the sole is slightly stroked. From the conditions just mentioned we have the characteristic "spastic gait"—dragging of the rigid and closely adducted legs, scraping the ground, tendency to walk on the toes, and tilting movement of the trunk at each step. As the process gradually ascends one finds as an early symptom an increase of the tendon reflexes in the arms. That is all—no disturbance of sensation, no Romberg's sign, no changes in the function of the bladder, bowel, or generative organs, no muscular atrophy, no change in their electrical reaction, no disturbance of the pupils, cerebral nerves, speech, psychical functions, memory, etc. The progress is usually very slow, but if the case should progress more rapidly, then the patient soon becomes altogether stiff and helpless; the arms become affected, and sometimes the back, neck, and head, the extreme contraction preventing any movement, so that the patients are in a most miserable plight and die marasmic. Generally, however, the disease is not directly fatal, and gives rise to less suffering than most of the chronic progressive spinal affections. The other disease to which Professor Erb had been able to differentiate was "syphilitic spinal paralysis." This occurs relatively early, that is during the first two to six years after infection, and in it, besides the typical picture of spastic paralysis as above described, there are always a disturbance of the bladder function, and usually a slight, but always demonstrable subjective and objective disturbance of sensation. The tendon reflexes are markedly increased, but the rigidity of the muscles is comparatively slight when contrasted with the apparently very marked spastic gait. There are no marked pains, no severe disturbance of the sensation, no atrophy of the muscles, and no disturbances of the brain or cerebral nerves. As to the pathological lesion at the bottom of the disorder it must be remembered that in syphilis, a disease presenting such an incredible number of different forms, variations are especially frequent, but as the result of many observations it appears that this particular form of disease in which spastic paralysis is combined with bladder trouble and disturbances of sensation is due to a combined system disease affecting chiefly the posterior half of the lateral tracts, the direct cerebellar tracts and the tracts of Gowers; then the posterior tracts, the tracts of Goll, and partly also those of Burdach. The point of practical importance is that these cases, in which in addition to the symptoms of spastic paralysis there are disturbances of sensation and of bladder function, are generally associated with antecedent syphilis, and that in their management energetic and repeated specific treatment with mercury and potassium iodide should have a place.

